# Updates on developing and applying biosensors for the detection of microorganisms, antimicrobial resistance genes and antibiotics: a scoping review

**DOI:** 10.3389/fpubh.2023.1240584

**Published:** 2023-09-07

**Authors:** Roberta Magnano San Lio, Martina Barchitta, Andrea Maugeri, Maria Clara La Rosa, Giuliana Favara, Antonella Agodi

**Affiliations:** Department of Medical and Surgical Sciences and Advanced Technologies “GF Ingrassia”, University of Catania, Catania, Italy

**Keywords:** antimicrobial resistance, antibiotic, antimicrobial resistance gene, biosensor, environment, clinical samples, public health

## Abstract

**Background:**

The inappropriate use of antibiotics in clinical and non-clinical settings contributes to the increasing prevalence of multidrug-resistant microorganisms. Contemporary endeavours are focused on exploring novel technological methodologies, striving to create cost-effective and valuable alternatives for detecting microorganisms, antimicrobial resistance genes (ARGs), and/or antibiotics across diverse matrices. Within this context, there exists an increasingly pressing demand to consolidate insights into potential biosensors and their implications for public health in the battle against antimicrobial resistance (AMR).

**Methods:**

A scoping review was carried out to map the research conducted on biosensors for the detection of microorganisms, ARGs and/or antibiotics in clinical and environmental samples. The Preferred Reporting Items for Systematic reviews and Meta-Analyses extension for Scoping Reviews (PRISMA-ScR) checklist was used. Articles published from 1999 to November 2022 and indexed in the following databases were included: MEDLINE, EMBASE, Web of Science, BIOSIS Citation index, Derwent Innovations index, and KCI-Korean Journal.

**Results:**

The 48 studies included in the scoping review described the development and/or validation of biosensors for the detection of microorganisms, ARGs and/or antibiotics. At its current stage, the detection of microorganisms and/or ARGs has focused primarily on the development and validation of biosensors in clinical and bacterial samples. By contrast, the detection of antibiotics has focused primarily on the development and validation of biosensors in environmental samples. Asides from target and samples, the intrinsic characteristics of biosensors described in the scoping review were heterogenous. Nonetheless, the number of studies assessing the efficacy and validation of the aforementioned biosensor remained limited, and there was also a lack of comparative analyses against conventional molecular techniques.

**Conclusion:**

Promoting high-quality research is essential to facilitate the integration of biosensors as innovative technologies within the realm of public health challenges, such as antimicrobial resistance AMR. Adopting a One-Health approach, it becomes imperative to delve deeper into these promising and feasible technologies, exploring their potential across diverse sample sets and matrices.

## 1. Introduction

Globally, the issue of antimicrobial resistance (AMR) is a significant public health concern, ranking among the top ten threats worldwide. Within the European Union alone, it is responsible for approximately 670,000 infections each year, leading to nearly 33,000 deaths (1). AMR predominantly emerges through bacterial mutations, driven by the selective pressure imposed by antibiotics, affording mutated strains a distinct competitive edge. The abuse of antibiotics, both in healthcare facilities and within communities, contributes to the spread of AMR, particularly in high-income countries (2). In healthcare settings, the transmission of multidrug-resistant microorganisms (MDR), such as carbapenem-resistant Enterobacteriaceae and methicillin-resistant *Staphylococcus aureus* (MRSA), significantly contributes to the prevalence of healthcare-associated infections (HAIs), with severe consequences for vulnerable patients (3). This situation is further aggravated by uncontrolled antibiotic utilization in non-clinical domains, including agriculture, aquaculture, and intensive farming, which accounts for a four-time higher volume than that observed in human usage (4). Notably, wastewater serves as a reservoir for antibiotic-resistant genes (ARGs) (5), considered as emerging pollutants in soil (6) and water (7). Despite wastewater treatment efforts, AMR bacteria and ARGs are not entirely eliminated, resulting in their discharge into the surrounding environment (8). The above-mentioned factors encourage the development of urgent strategies to detect and characterize microorganisms, ARGs and antibiotics in a wide range of matrixes and settings. Nowadays, molecular methods and metagenomic analyses partially supports this need (9–13). Traditional methods for diagnosing bacterial infections involve several approaches, including cell culture, polymerase chain reaction (PCR) to identify specific nucleic acids, and immunological techniques such as enzyme-linked immunosorbent assay (ELISA) that rely on antigen–antibody interactions (14). Nevertheless, traditional approaches might incur significant costs and time requirements, often involving intricate, multi-step sample preparations. Ongoing efforts are underway to explore novel technological approaches that seek to create affordable and effective alternatives for diagnosing bacterial infections. These approaches aim to provide rapid results and can be conveniently deployed at the point of care (15).

**Figure 1:**
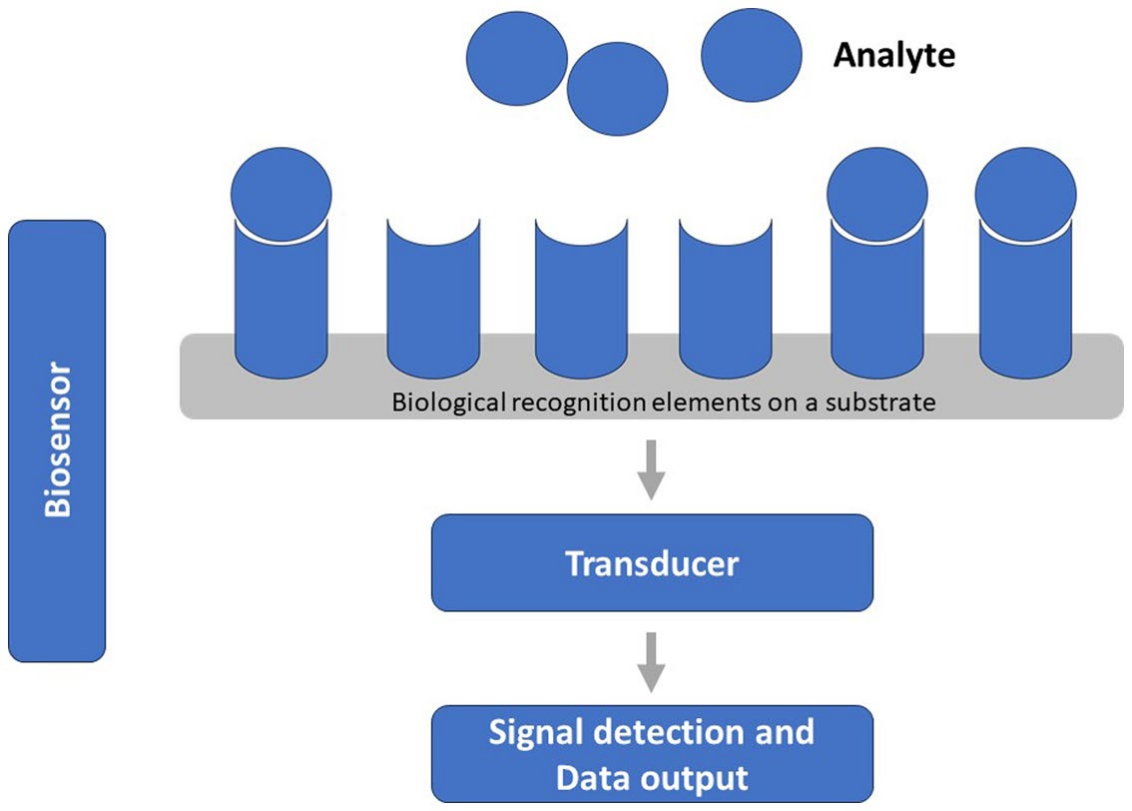
General characteristics and components of a biosensor.

In this scenario, a typical biosensor is able of detecting chemical compound by means of a biological component, usually represented by biomolecule, whole cells, fragments of biological tissue or bacteria. The main three components of biosensors are characterized by the biological recognition element (e.g., antibodies, enzymes, DNA, RNA, and cells) linked to a substrate (e.g., silicon, gold, glass, and polymers) and integrated with a transducer that produces a signal to transform the interaction with the target into a more easily measurable and quantifiable signal (16) (Figure 1). More precisely, the chemical reaction generates a signal (such as fluorescence, heat generation, alterations in colour, oscillatory frequency, or conductivity), which is then translated into a measurable indication that correlates directly with the magnitude of the signal. Regarding inherent attributes, biosensors rely on various methodologies, including electrochemical methods (16), encompassing direct or alternating current-based approaches, as well as mechanical techniques (such as nanoparticle-functionalized piezoelectric biosensors) and optical methods (e.g., optical fibers, surface plasmon resonance, and luminescence) (17, 18). Key requisites for devising a practical and user-friendly tool encompass the need for a biosensor to exhibit a significant level of specificity and sensitivity, robust stability under operational parameters (such as temperature and pH), as well as swift response time and a low limit of detection (18). In recent years, several studies have proposed the development and validation of biosensors for the detection of microorganisms (e.g., *S. aureus* and *Mycobacterium tuberculosis*), as well as ARGs or antibiotics, in different clinical and environmental samples. On one hand, biosensors for the detection of microorganisms or resistance genes have been validated in clinical and/or bacterial samples (19). On the other hand, biosensors for the detection of antibiotics have been mostly validated in environmental samples (e.g., milk and meat samples, animal feces, and serum) (20). Although the crucial role of water in the increased spread of AMR, only few studies focused on water samples, suggesting the need to develop specific biosensors. Furthermore, there is a growing interest in the advancement of biosensors based on aptamers. Aptamers are single-stranded oligonucleotides that are meticulously chosen from combinatorial libraries due to their exceptional ability to bind with high affinity to specific target molecules (18, 21). While several systematic reviews exist about the development of biosensors in the field of AMR, none of them have simultaneously summarized the various aspects concerning the type of target considered and the potential application in clinical, bacterial, and environmental samples. A scoping review was therefore carried out to map the research conducted on biosensors for the detection of microorganisms, antimicrobial resistance genes and/or antibiotics in different matrixes. Consequently, the primary focus of this study revolved around exploring existing literature to ascertain the current understanding of biosensors as cutting-edge technologies and their potential applications.

## 2. Materials and methods

For the present scoping review, the Preferred Reporting Items for Systematic Reviews and Meta-Analyses extension for Scoping Reviews (PRISMA-ScR) checklist was employed (9). To ensure comprehensive coverage of relevant literature, a search was conducted across the following databases, covering the period from 1999 to November 2022: MEDLINE, EMBASE, Web of Science, BIOSIS Citation index, Derwent Innovations index, and KCI-Korean Journal database. The search strategy consisted of the following combination of terms: (Biosensors OR Biosensor OR Bioprobes OR Bioprobe) AND (Drug Resistances, Microbial OR Antimicrobial Drug Resistance OR Antimicrobial Drug Resistances OR Antibiotic Resistance OR Antibiotic Resistances).

To be included in the scoping review, articles needed to report studies using biosensors to collect data about the presence of microorganisms, ARGs and antibiotics in clinical and/or environmental samples. Studies were selected only if they: (i) were written in English, (ii) included the development and validation of biosensors for the detection of microorganisms and/or genes of resistance and/or antibiotics, and (iii) using clinical and/or bacterial and/or environmental samples. Articles that did not align with the primary focus of the scoping review were excluded. Additionally, abstracts, editorials, commentaries, reviews, systematic reviews, and meta-analyses were also excluded from the study.

The study selection and data charting processes were conducted independently by two authors using a standardized data abstraction tool specifically developed for this scoping review. The tool systematically captured essential details about the articles, including: (i) author information, (ii) publication year, (iii) country of origin, (iv) comprehensive information about the sensors employed (e.g., sensor type, target, and sample types), and (v) the key findings obtained. In the event of any disagreements, they were resolved through discussion between the two authors or, if necessary, by involving a third author for further adjudication.

Studies were grouped by the type of target considered (i.e., detection of microorganism, antimicrobial resistance genes and/or antibiotics), and summarized describing the development and application of these biosensors in clinical, bacterial and environmental samples.

## 3. Results

### 3.1. Study selection

**Figure 2:**
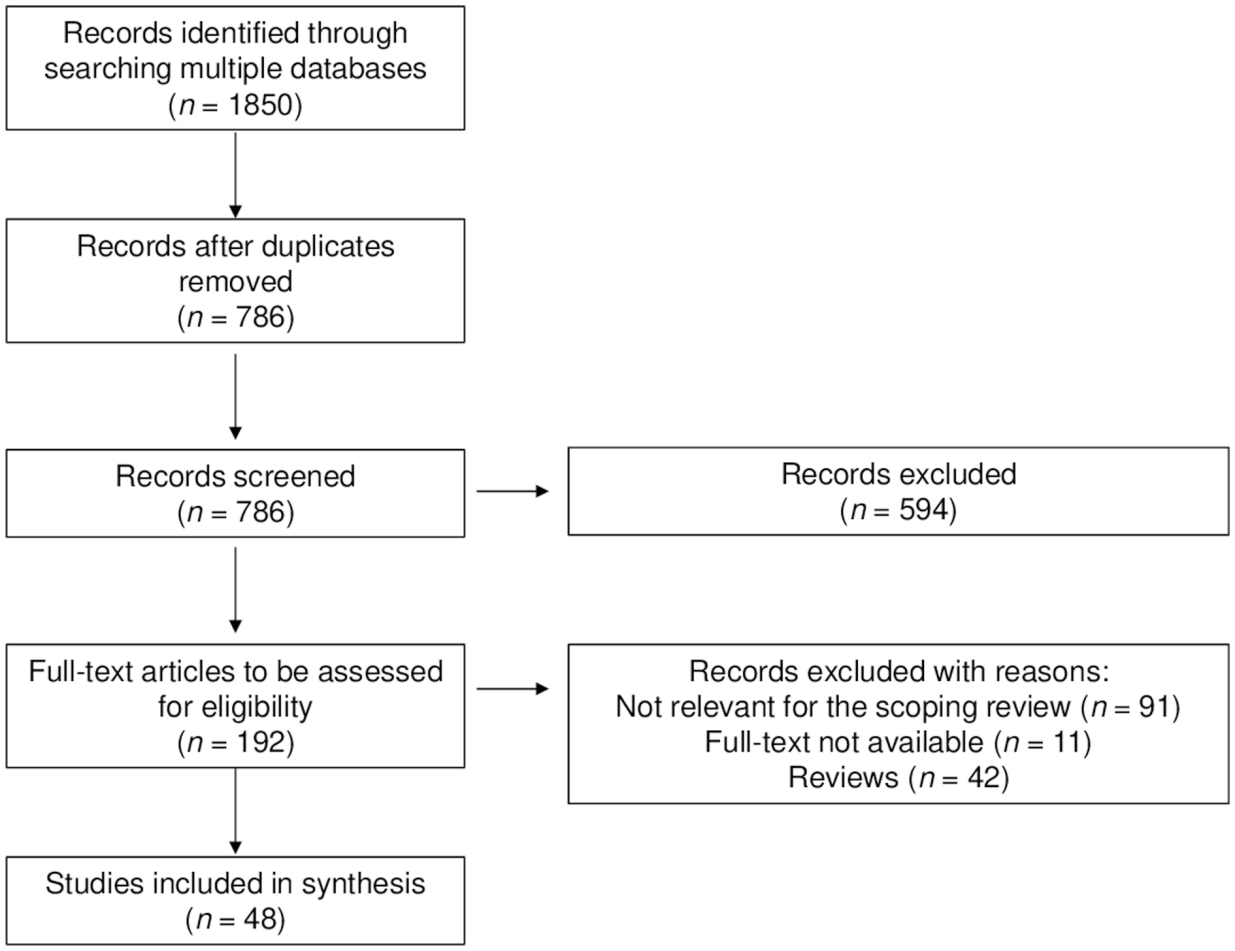
Selection of studies included in the scoping review.

Following the removal of duplicate articles, a comprehensive search of literature databases yielded a total of 786 unique articles. Through the screening of titles and abstracts, 594 articles were excluded. Subsequently, 192 full-text articles were retrieved and assessed for eligibility. Out of these, 144 articles were excluded for various reasons: 91 were not relevant for the objective of the current scoping review, 11 lacked full-text availability, and 42 were identified as reviews. Finally, a total of 48 studies met the eligibility criteria and were included in this scoping review (Figure 2).

### 3.2. Development and validation of biosensors for the detection of microorganisms and antimicrobial resistance

#### 3.2.1. Characteristics of the studies

In our scoping review, we included 25 studies aimed to develop and/or validate biosensors for the detection of ARGs and/or AMR microorganisms. Tables 1, 2 show the main characteristics of biosensors developed for clinical and bacterial samples, respectively. Overall, the studies were conducted within the timeframe spanning from 1999 to 2022. Among them, 6 studies were conducted in the United States of America (22–27), 8 studies in China (28–35), while 2 studies were conducted in Canada (36, 37) and UK (38, 39), respectively. Only 1 study was conducted in Australia (40), Italy (41), Spain (42), France (43), Switzerland (44), Thailand (45) and Turkey (46), respectively. Across these studies, a diverse array of biosensors was delineated by the authors, with a variety of samples employed for validation. Notably, the authors of the encompassed studies characterized their biosensors as: silicon-based biosensors (22, 23, 25); based on nanoscale film on optical fiber (26); surface plasmon resonance biosensor (37); sensitive surface-enhanced Raman scattering biosensor (35); biosensors based on colorimetric assays (30, 32) or on new nanocomposite materials (43); binary deoxyribozyme biosensor (27); electric and electrochemical biosensors (24, 38–41, 44–46); electrochemical aptasensor (34); conventional multiple cross displacement amplification technique combined with nanoparticle-based lateral flow biosensor (29, 31, 33), ultrasensitive nanophotonic bimodal waveguide interferometer (42), and biochip systems (28, 36). Regarding the specimens employed in biosensor development and/or validation, 9 studies worked on clinical samples (e.g., human serum and urine samples) (22, 25, 26, 28, 35, 36, 38, 44, 46), while 13 on bacterial samples (23, 24, 29, 30, 32, 33, 37, 39–43, 45). Three studies used both clinical and bacterial samples (27, 31, 34).

**Table 1:** Summary of studies on biosensors for the detection of microorganisms and/or antibiotic-resistance genes in clinical samples.

Authors and year of publications	Country	Type of sensor	Transduction systems	Sample	Target	Parameters
Ostroff et al., 1999	USA	Silicon-based biosensor	Optical	Human serum and urine samples	To develop a silicon-based biosensor and to validate it through the detection of six *mec*A target molecules in different clinical samples	Limit of detection (LoD) = 5 pmol/L (150 pmol/sample)
Lindsey et al., 2008	USA	Silicon-based biosensor	Optical	Blood culture	To detect *tuf* gene for the identification of *Staphylococcus* genus, *femB* gene for the identification of *S.aureus* species, and *mecA* gene for the identification of methicillin- resistance	LoD = 5 × 10^7^ CFU/mL
Kara et al., 2008	Turkey	Electrochemical genosensor	Electrochemical	Bacterial DNA	To detect multiple point mutations in *rpo*B gene of *M. tuberculosis* during the development of rifampicin resistance	LoD = 18.65–21.48 fmol/mL
Guo et al., 2009	China	Biochip system	Optical	Bacterial cultures and sputum specimens	To develop and evaluate a rapid biochip system for the determination of multidrug-resistant tuberculosis in *M. tuberculosis* isolates and clinical sputum samples	LoD = the lowest concentration of *M. tuberculosis*
Lam et al., 2013	Canada	Solution-based circuits formed on chip	Electrochemical	Urine samples	To yield rapid and accurate information on microorganisms and antibiotic resistance of pathogens	LoD: 1 cfu μl−1
Huang et al., 2015	UK	Electrochemical biosensor	Electrochemical	Bacterial DNA	To detect bla NDM, the gene encoding the emerging New Delhi metallo-beta-lactamase,	LoD = 10 nM for synthetic targets; LoD = 100 pM for PCR products
Bandara et al., 2011	USA	Nanoscale-film optical fiber sensor (NOFS)	Optical	Bacterial culture	To detect methicillin-resistance among *Staphylococcus* species	NA
Hu et al., 2019	China	Multiple cross displacement amplification (MCDA) combined with lateral flow biosensor (LFB)	Optical	Bacterial DNA and sputum samples	To detect all *A. baumannii* strains and to identify the strains harboring bla OXA-23-like gene	LoD = 100 fg templates per tube for pgaD and blaOXA-23-like genes in pure cultures
Bengtson et al., 2017	USA	Binary deoxyribozyme (BiDz) sensors (TB-DzT)	Optical	Bacterial DNA	To detect *M. tuberculosis* and resistance to multiple antibiotic	LoD = 5 fg – 15.6 pg. of Mtb CDC1551 DNA
Wang et al., 2018	China	Sensitive surface-enhanced Raman scattering (SERS) biosensor	Optical	Bacterial culture	To capture and discriminate bacterial pathogens from solution	LoD = 5 × 102 cells/mL
Suea-Ngam et al., 2021	Switzerland	Electrochemical CRISPR/Cas biosensor utilizing silver metallization (termed E-Si-CRISPR)	Electrochemical	Human serum and bacterial culture	To detect MRSA	NA
Wei et al., 2022	China	Aptamer-based colorimetric = biosensor	Optical	Bacterial DNA	To develop a biosensor for the detection of MRSA	NA

**Table 2:** Summary of studies on biosensors for the detection of microorganisms and/or antibiotic-resistance genes in bacterial samples.

Authors and year of publications	Country	Type of sensor	Transduction systems	Sample	Target	Parameters
Jenison et al., 2000	USA	Silicon-based biosensor	Optical	Bacterial DNA	To describe a silicon-based biosensor and validate it through the detection of *mec*A gene present in methicillin-resistant *S. aureus*	LoD = 1 pM
Mac Sweeney et al., 2004	USA	Optical DNA hybridization biosensor	Optical	Bacterial DNA	To develop an optical DNA hybridization biosensor based on ruthenium electrochemiluminescence to detect DNA from the *rpo*B gene of *M. tuberculosis*	NA
Tombelli et al., 2005	Italy	DNA-based piezoelectric biosensor	Mechanical	Bacterial DNA	To develop and validate a DNA-based piezoelectric biosensor for the detection of the *mec*A gene of methicillin-resistant *S. aureus* (MRSA)	NA
Wang et al., 2018	China	Conventional multiple cross displacement amplification (MCDA) technique combined with nanoparticle-based lateral flow biosensor (MCDA-LFB)	Optical	Bacterial DNA	To detect all *S. aureus* strains, and to differentiate MRSA from methicillin-sensitive *S. aureus*	LoD = 100 fg DNA/reaction for nuc and mecA genes in the pure cultures; LoD = 10 CFU/tube for nuc and mecA genes in the blood samples
Rachkov et al., 2013	Canada	Surface plasmon resonance (SPR) biosensor	Optical	Bacterial DNA	To detect single base mismatched oligonucleotides related to the *rpo*B gene of *M. tuberculosis*	NA
Sun et al., 2019	China	Colorimetric- and electrochemical-based bioassay	Electrochemical	Bacterial culture	To develop a p-Benzoquinone-mediated bioassay for the detection of *E.coli* and its relative level of antibiotic resistance	Concentration ranges = 1.0 × 10^3^ to 1.0 × 10^9^ CFU/mL
Maldonado et al., 2020	Spain	Ultrasensitive nanophotonic bimodal waveguide interferometer (BiMW)	Optical	Bacterial DNA	To detect two prevalent and clinically relevant Gram-negative antimicrobial resistance encoding sequences: the extended-spectrum beta lactamase-encoding gene blaCTX-M-15 and the carbapenemase-encoding gene blaNDM-5	LoD = ~10^5^ CFU mL^−1^
Abeyrathne et al., 2016	Australia	Biosensor based on interdigitated electrodes	Electrochemical	Bacterial culture	To detect *S. aureus* and ascertain its sensitivity to flucloxacillin rapidly	NA
Schulze et al., 2021	UK	Label-Free Electrochemical Sensor	Electrochemical	Bacterial culture	To combine a new class of non-biological binder molecules with electrochemical impedance spectroscopy (EIS)-based sensor detection for direct, label-free detection of Gram-positive bacteria	Optical Density 600 range = 0.002–0.1
Bizid et al., 2018	France	Direct DNA sensor based on new nanocomposite materials (Fc-ac-OMPA/MWCNTs)	Electrochemical	Bacterial DNA	To detect DNA of the *rpo*B gene of *M. tuberculosis* in real PCR samples	LoD = 0.08 fmol L^−1^
Zhou et al., 2022	China	Label-free visible colorimetric biosensor	Optical	Bacterial culture	To develop a colorimetric biosensor for the detection of multiple pathogenic bacteria based on engineered polydiacetylene liposomes	Bacterial concentration = 1–10^10^ CFU/mL
Pintavirooj et al., 2022	Thailand	Molecularly Imprinted Polymer (MIP)-based electrochemical biosensor	Electrochemical	Bacterial culture	To develop a MIP-based electrochemical biosensor for the detection of *K. pneumoniae*	NA
Chen et al., 2020	China	Multiplex loop-mediated isothermal amplification linked to a nanoparticle-based lateral flow biosensor (m-LAMP-LFB)	Optical	Bacterial DNA	To detect all *S. aureus* species and to identify MRSA	LoD = 100 fg of genomic DNA template per reaction for femA and mecA detection

#### 3.2.2. Biosensors in clinical samples

Overall, 9 studies described the development and/or validation of biosensors for the detection of microorganism and/or ARGs in clinical samples (22, 25, 26, 28, 35, 36, 38, 44, 46). Traditionally, nucleic acid hybridization has been analysed using colorimetric, chemiluminescent, radioactive, or fluorescent labels. Nevertheless, it has been demonstrated that biosensors are capable of detecting nucleic acid hybridization even in the absence of labelled reporter molecules. For example, Ostroff and colleagues developed a silicon-based biosensor that generated a visual signal in response to nucleic acid targets by directly interacting with light and thin films on the modified silicon surface. The surface of the biosensor was adapted using capture oligonucleotide probes. Through the hybridization process with complementary targets and biotinylated detector oligonucleotides, this modification facilitated the creation of an organic thin film. Subsequent enhancement through peroxidase led to the deposition of an insoluble product on the silicon surface. Modifications in the patterns of reflected light interference induced a transition in colour from gold to purple. This change correlated with variations in surface thickness influenced by the hybridization of target molecules. Importantly, this biosensor exhibited the capability to detect a diverse range of targets, including single-nucleotide polymorphisms, within human serum and urine samples. Consistent with this, six synthetic *mec*A target molecules were generated to evaluate the discrimination capacity of the biosensor, which was subsequently validated. The outcomes can be acquired swiftly, within a concise span of 25 min, through direct visual examination. Alternatively, quantification can be attained employing ellipsometry. The biosensor demonstrates a remarkable detection limit of 5 pmol/L (22). Likewise, Lindsey and colleagues employed a silicon-based biosensor to detect the *mec*A gene, enabling the identification of methicillin resistance. They also targeted the *tuf* and *fem*B genes to distinguish the *Staphylococcus* genus and species, respectively. The authors tested 107 consecutive positive blood cultures of clinical patients, and the assay exhibited 100% concordance with standard microbiological methods. The process of target immobilization initiated a sequence of reactions that transformed hybridization events into a molecular thin film, inducing a transition in surface colour from gold to blue. Impressively, results were generated within just 90 min directly from signal-positive samples, all without the requirement for any instrumentation (25). Due to the rising methicillin-resistance among *Staphylococcus* species in hospitals, communities and animals, it should be necessary to develop culture-free diagnostic assays. In such a context, optical fibers employed as biosensors offer a multitude of advantages, notably encompassing their lightweight nature, cost-effectiveness, and capacity to capture emitted light from targets post-excitation. Bandara and collaborators put forward the concept of a nanoscale-film optical fiber sensor tailored for the identification of methicillin-resistant staphylococci. The team engineered an optical fiber coated with a self-assembled nanoscale film, intricately linked with monoclonal antibodies specifically targeting penicillin-binding protein 2a (PBP2A). Given that PBP2A serves as a distinctive marker for methicillin-resistant staphylococci, samples were procured from healthy volunteers via swabs extracted from the ear canal, nostril, palm, or arm skin. Upon the binding of the target antigen with the monoclonal antibodies, alterations in the thickness and refractive index of the optical fiber’s thin film coating were instigated. These alterations yielded a discernible output, serving as a clear indicator of the presence of the target antigen. The results ascertained a promising system for rapid and specific detection of MRSA (26). Interestingly, Suea-Ngam and colleagues introduced an electrochemical CRISPR/Cas biosensor called E-Si-CRISPR, which utilized silver metallization for the detection of MRSA. This innovative biosensor, as described by the researchers, eliminated the need for nucleic acid amplification and demonstrated outstanding analytical performance. Notably, the biosensor exhibited remarkable selectivity for MRSA in human serum, even in the presence of other commonly encountered bacteria (44).

Beyond *S. aureus*, the rising prevalence of drug-resistant *M. tuberculosis* strains is widely acknowledged. In this context, Kara and collaborators introduced an innovative electrochemical genosensor technology designed for the direct identification of numerous point mutations within the *rpo*B gene. This technology harnessed bacterial DNA extracted from clinical isolates. As detailed, the genosensor centered on graphite electrode arrays that were tailored with five distinct guanine-free aminohexyl-tethered oligonucleotide probes. These probes exhibited the capability to identify various segments within the hotspot region of the bacterial *rpo*B gene. The resulting electrochemical signal, stemming from fluctuations in peak voltage—specifically linked to guanine oxidation—served as a reliable indicator of the hybridization process between PCR amplicons and inosine-modified capture probes situated on the graphite surface. This electrochemical signal proved valuable for detecting the presence of mutations that confer rifampicin resistance (46). Interestingly, Guo and colleagues developed a rapid biochip system to simultaneously identify the most frequent mutations in the *rpo*B, *kat*G and *inh*A genes in *M. tuberculosis* isolates and clinical sputum samples. The biochip was developed, featuring covalently immobilized oligonucleotide probes on the slides. The biochip itself comprised four distinct arrays. Following the multiplex asymmetric polymerase chain reaction and subsequent biochip hybridization, dedicated software was employed to quantify fluorescent intensities. When juxtaposed against conventional susceptibility testing and DNA sequencing, the biochip exhibited remarkable agreement with both approaches, delivering a straightforward and precise clinical assay that could be completed within a mere six-hour timeframe (28). In order to overcome the necessity of employing an active multiplexing strategy for highly multiplexed arrays, Lam and colleagues introduced a novel approach utilizing a two-dimensional array of electrodes. This approach allowed for the programming of transient solution-based circuits, enabling individual analysis of sensors while utilizing shared contacts. Relative to the incorporation of expensive active electronics onto silicon surfaces, this approach presented notable advantages. The solution-based circuit comprised 100 working electrodes, 30 off-chip contacts, and 5 liquid channels, with each channel containing 20 sensors. Notably, the authors successfully adapted the chip’s multiplexing capabilities to detect various pathogens and investigate antibiotic resistance, broadening its potential applications. To develop and validate the system, the authors generated a list of potential probes based on the *rpo*B gene sequences of the bacteria under study. The set of probes designed for mRNA targets, which imparts pathogen specificity to individual sensors, encompassed 90% of prevalent urinary tract pathogens. These probes exhibited the capability to concurrently identify multiple analytes, including clinically relevant levels of *Escherichia coli*. Furthermore, the system demonstrated compatibility with unpurified bacterial lysates and samples spanning microliters to milliliters. Given these merits, this technology holds the potential to mark a substantial advancement in the realm of biomolecular sensing (36).

Within the realm of optical biosensors, Surface-Enhanced Raman Scattering (SERS) has emerged as a compelling avenue for bacteria detection, owing to its exceptional sensitivity and straightforward setup. Aligning with this, Wang and collaborators introduced a biosensor founded on this principle. The biosensor exhibited impressive bacterial-capture efficacy (65%) across various pathogens within complex solutions (such as *E. coli*, *S. aureus*, and MRSA), all while maintaining a brief assay duration (30 min). Furthermore, the efficacy of this approach was successfully demonstrated in both milk and blood samples (35).

Due to the escalating prevalence of MDR Gram-negative pathogens, researchers have been driven to develop innovative biosensors capable of detecting key resistance genes involved in this phenomenon. For instance, Huang and colleagues focused on the development of an electrochemical biosensor to detect blaNDM, the gene responsible for encoding the emerging New Delhi metallo-beta-lactamase. Their study involved the use of a clinical isolate, specifically the NDM-1 producing *Citrobacter freundii strain*. The researchers employed *in silico* techniques to design blaNDM-specific nucleic acid probes, which were subsequently immobilized on gold screen-printed electrodes. The biosensor utilized label-free electrochemical impedance spectroscopy, aiming to detect blaNDM PCR products and achieve direct, label-and amplification-free detection of blaNDM plasmid DNA. The effectiveness of the assay was successfully demonstrated through the detection of synthetic targets, PCR products, as well as direct amplification-free detection from a blaNDM-harboring plasmid (38).

#### 3.2.3. Biosensors in bacterial samples

Collectively, a total of 13 studies detailed the creation and/or validation of sensors directly aimed at detecting microorganisms and/or ARGs within bacterial samples. Furthermore, 3 study described the development and/or validation of these sensors in both clinical and bacterial samples (27, 31, 34). In relation to studies exploring sensors for the detection of microorganisms and/or genes of resistance in bacterial samples, some authors employed bacterial DNA (23, 24, 29, 33, 37, 41–43), while others bacterial cultures (30, 32, 39, 40, 45).

Following in the footsteps of Ostroff and Lindsey, Jenison and colleagues developed a silicon-based biosensor engineered to identify the *mec*A gene. By utilizing DNA obtained from a MRSA strain, the researchers demonstrated how enzymatic transduction facilitated hybridization, resulting in the formation of a molecular thin film that was visibly detectable under white light. Initially, at a surface thickness of 0 Å (indicating an unreacted surface), the colour appeared as gold. However, as the surface thickness increased by 100 Å, the colour transitioned into the red range and further progressed to purple and white, eventually approaching its original gold hue. The colour signal was successfully detected when DNA isolated from a MRSA strain was utilized, whereas no colour signal was observed in the case of a *S. aureus* strain lacking the *mec*A gene (23). In line, Tombelli and colleagues described the development of a biosensor for detecting the *mec*A gene in MRSA. However, the authors proposed a mechanical DNA-based piezoelectric biosensor, in which the detection of hybridisation relied on frequency changes derived from the interaction between a specific probe and its complementary strand. The probe was immobilized on a gold electrode situated on a quartz crystal. The authors specifically compared two methods of probe immobilization: one involved streptavidin-biotin interaction, while the other involved direct immobilization of thiolated probes. By evaluating these two approaches, the researchers aimed to determine the most effective and efficient method for immobilizing probes specific to the *mecA* gene Moreover, the authors compared two pre-treatments of bacterial DNA with different denaturation procedures. The best results were obtained with the immobilization of biotinylated probe and with PCR-amplified samples treated with the thermal denaturation involving blocking oligonucleotides. Therefore, with appropriate optimization of PCR conditions, the proposed biosensor holds potential for broader application in detecting PCR-amplified DNA from actual clinical samples (41). Unlike prior studies, Wei and colleagues proposed a biosensor based on aptamers, defined as short oligonucleotide molecules that, unlike their natural counterparts (i.e., DNA and RNA), offer distinct advantages in terms of their selectivity and binding properties. The aptamer-based colorimetric biosensor used the CRISPR/Cas12a system and recombinase polymerase amplification to identify MRSA. Their results, also confirmed by the analyses of clinical samples, revealed the great potential of the biosensor as robust antibiotic-resistant bacteria detection platform (34). In a similar context, Wang and collaborators pursued the development of a biosensor intended for the detection of *S. aureus* while also discerning methicillin resistance. However, the biosensor they devised relies on the conventional multiple cross displacement amplification technique (MCDA). The proposed approach combines the MCDA assays, known for their high selectivity in target sequence detection through a specific set of 10 primers, with a nanoparticle-based lateral flow biosensor (MCDA-LFB). A comprehensive evaluation of the assay was conducted using 58 strains, including various species of Gram-positive and Gram-negative strains. The results demonstrated a remarkable analytical specificity of 100% and no cross-reactivity to non-*S. aureus* strains during specificity testing. These findings highlight the potential of the biosensor as a simple, rapid, and reliable method for detecting all *S. aureus* strains and identifying MRSA infections, enabling appropriate antibiotic therapy decisions (29). With the same aim, Chen and colleagues proposed a multiplex loop-mediated isothermal amplification coupled with a nanoparticle-based lateral flow biosensor (m-LAMP-LFB) for the detection of all species of *S. aureus* and MRSA. The primers were designed based on the *femA* gene (a gene specific to *S. aureus*) and the *mecA* gene. The multiple-LAMP products were analysed using a colorimetric indicator and a lateral flow biosensor. Notably, the amplification process resulted in a colour change from colourless to light green, facilitating visual detection. The authors also described the optimization of the assay and confirmed its exceptional specificity of 100%. Considering these advantages, the developed biosensor holds promise as a reliable method for the rapid detection of *S. aureus* and MRSA, even in clinical samples (33). While the previous authors described the MCDA-LFB for the detection of *S. aureus*, Hu and colleagues proposed this biosensor for detecting all *Acinetobacter baumannii* strains and its carbapenem-resistant gene *bla* OXA-23-like, using bacterial strains from the American Type Culture Collection and Shougang Hospital. The authors validated the optimal reaction condition and the specificity of the assay, which was of 100%. Interestingly, the application of the MCDA-LFB assay was evaluated using clinical samples (i.e., DNA templates extracted from 110 sputum samples), showing consistent results with conventional culture-based technique (31).

Growing interest there has been also for the development of biosensor for the detection of *M. tuberculosis* and antibiotic resistant strains, as suggested by two studies (24, 43). Mac Sweeney and colleagues presented an optical DNA hybridization biosensor utilizing ruthenium electrochemiluminescence for detecting DNA from the *rpo*B gene of *M. tuberculosis*. The target DNA strand was specifically labelled with a ruthenium ester, which, upon hybridization, emitted light at a wavelength of 620 nm. The biosensor employed a solid-state silicon PIN photodiode as the transduction element. The development process focused on characterizing and optimizing each component of the biosensor to maximize sensitivity for detecting the electrochemiluminescent optical signal generated during the DNA hybridization event (24). The biosensor developed by Bizid and colleagues, instead, was a direct DNA sensor based on new nanocomposite materials (Fc-ac-OMPA/MWCNTs). According to the authors, the introduced nanocomposite membrane in this biosensor enhanced the anchoring of DNA strands, as evidenced by the improved electrochemical response compared to the biosensor based solely on the redox oligomer. Notably, the integration of PCR amplification with electrochemical devices offered a sensitive approach for detecting DNA from the *rpo*B gene of *M. tuberculosis*. The biosensor exhibited several favourable attributes, including ease of construction, excellent reproducibility, and minimal time requirements, making it highly competitive for future applications in pathogen diagnostics and therapeutics (43). In this scenario, Bengtson and colleagues described the binary deoxyribozyme sensors for the multiplex detection of extensively *M. tuberculosis* multidrug resistant in both clinical and bacterial samples (27). More precisely, the proposed sensor integrated multiplex PCR with the detection of single nucleotide polymorphisms – specifically targeting *rpo*B, *kat*G, *inh*A, and *gyr*A – employing highly selective binary deoxyribozymes. As a result, this sensor was designed to focus on genetic loci associated with resistance against rifampicin, isoniazid, and fluoroquinolone antibiotics. The authors adeptly showcased the sensor’s capability to accurately detect *M. tuberculosis* along with five mutations linked to resistance against the aforementioned three antibiotics, all within clinical isolates. Moreover, the assay exhibited the potential to identify not only a minor population of drug-resistant *M. tuberculosis* strains but also previously unknown mutations at the targeted genetic loci (27). As proposed by Rachkov and colleagues, a surface plasmon resonance biosensor system could effectively detect single base mismatched oligonucleotides associated with the *rpo*B gene of *M. tuberculosis*. The authors elaborated on the creation of the instrument, the immobilization of probes, and the subsequent target hybridization process. Their study primarily delved into the hybridization dynamics between an immobilized probe and its counterparts – both perfectly matched and single base mismatched targets. This analysis was performed in buffer solutions with varying stringencies. The results demonstrated distinct levels of hybridization between the two target types and the immobilized probe, enabling the discrimination between perfectly matched and single base mismatched targets. The ability to achieve optimal discrimination and a rapid response time holds potential for the identification of highly virulent microbial organisms through DNA hybridization. Additionally, the surface plasmon resonance technology could be utilized to expand the capabilities of the sensor, potentially creating an array that is valuable for antimicrobial resistance profiling of *M. tuberculosis* (37).

Other researchers directed their attention towards the growing necessity for swift and direct detection of the most significant Gram-negative antimicrobial resistance encoding sequences. Maldonado and colleagues introduced a novel biosensor methodology that utilizes an ultrasensitive nanophotonic bimodal waveguide interferometer for the direct detection of the blaCTX-M-15 and blaNDM-5 genes, without the need for amplification. This approach was based on the evanescent field principle, which enables the monitoring of local changes in the refractive index at the sensor surface. In addition, the researchers incorporated pre-analytical steps such as DNA extraction, fragmentation, and denaturation to facilitate the detection of gene DNA using the developed biosensor. The results demonstrated that the biosensor exhibited exceptional sensitivity and specificity, making it a valuable diagnostic tool for the rapid identification of drug resistance in less than 30 min. Given these capabilities, the biosensor holds promise for the control and management of healthcare-associated infections caused by multidrug-resistant bacteria (42).

Five studies described sensors for the detection of microorganism and/or ARGs using bacterial cultures (30, 32, 39, 40, 45). Zhou et al. documented the creation of a colorimetric biosensor employing engineered polydiacetylene liposomes to detect various pathogenic bacteria. The study utilized *Pseudomonas aeruginosa*, *S. aureus*, *E. coli*, *Streptococcus pneumoniae*, *Klebsiella pneumoniae*, and *Enterococcus faecalis*, sourced from reputable repositories such as ATCC or CMCC (National Center for Medical Culture Collections). The remarkable variation in chromatic colour alteration observed in their research was attributed to the distinct interactions occurring between bacterial toxins and biomimetic lipid bilayers. The researchers developed a colorimetric fingerprint array to distinguish between different microorganisms by examining the interaction between bacterially secreted toxins and liposome bilayers. The label-free liposome-based system exhibited superior response time and sensitivity compared to other chromatic bacterial detection assays. Interestingly, the authors found that even without any ligand conjugation, liposomes were still capable of accurately distinguishing between pore-formation haemolysis and phospholipase haemolysis with relatively high sensitivity (32).

In this context, Sun and colleagues proposed a colorimetric and electrochemical-based bioassay for the detection of *E. coli* – using the p-benzoquinone as a redox mediator – and its relative level of antibiotic resistance. A visible colour change – captured with a smartphone using a “light box” – could reflect the concentration of bacteria. Furthermore, the practicality of the bioassay in detecting antibiotic-resistant bacteria was demonstrated using two artificially induced antibiotic-resistant bacterial strains. In contrast to existing electrochemical-based bacterial biosensors, the authors highlighted that their assay eliminated the need for additional sample processing steps, enzymes, and complex instruments to visualize the results. For these reasons, the assay could be a rapid and cost-effective method to identify *E. coli* and its resistance (30).

Abeyrathne and colleagues, instead, proposed a chip sensor to detect *S. aureus* and ascertain its sensitivity to flucloxacillin rapidly. The biosensor was based on interdigitated electrodes and measured the impedance change caused by binding of bacteria to antibodies directed against *S. aureus* immobilized on the surface. Furthermore, the biosensor assessed the altered conductivity of the medium caused by bacterial growth. Precisely, the electrical measurements of the sensor effectively distinguished positive from negative control samples within an hour and detected flucloxacillin sensitivity within 2 h. These results provided motivation for the continued advancement of the sensor for application in whole blood and other bacterial subtypes (40).

Additionally, Pintavirooj and colleagues introduced a novel approach using a molecularly imprinted polymer-based electrochemical biosensor for detecting *K. pneumoniae*. The analysis employed cyclic voltammetry detection methods based on monitoring current variations at the sensor surface following biochemical interactions on the electrode. The biosensor allowed for quantitative detection by employing an electrochemical technique to measure alterations in electrical signals across various concentrations of *K. pneumoniae*. Moreover, the sensitivity test showed that biosensor for *K. pneumoniae* detection with a gold electrode was the most sensitive, if compared to biosensor applied to other microorganisms (i.e., *P. aeruginosa* and *E. faecalis*). Thus, the sensor was easy to adopt, with high accuracy and specificity for *K. pneumoniae* detection (45).

Furthermore, the detection of Gram-positive microorganisms has been investigated through the development of innovative biosensor. For instance, Schulze and colleagues integrated a novel class of non-biological binder molecules with an electrochemical impedance spectroscopy-based sensor. The aim was the direct, label-free detection of *Staphylococcus carnosus*, which caused the specific conformation changes of the polymers immobilized on the surface of gold screen-printed electrodes. Specifically, the detection of *S. carnosus* was achieved within a short incubation period of only 20 min when the sample solution was exposed to the polymer-functionalized electrodes. Notably, the results demonstrated a distinct differentiation between Gram-positive and Gram-negative bacteria, representing a notable improvement over current bacterial detection assays (39).

### 3.3. Development and validation of biosensors for the detection of antibiotics

#### 3.3.1. Characteristics of studies included

In our scoping review, we included 23 studies aimed to develop and/or validate biosensors for the detection of antibiotics. Tables 3, 4 show the main characteristics of biosensors developed for clinical/bacterial samples and environmental samples, respectively. In general, studies were conducted from 2002 to 2021. Among them, 3 studies were conducted in the United States of America (47–49), 3 in Germany (50–52), 5 in China (53–57), 2 in Switzerland (58, 59) and India (60, 61), respectively. Only 1 study was conducted in UK (62), Denmark (63), New Zeeland (64), Japan (65), Taiwan (66), Iran (67), Australia (68), and Saudi Arabia (69), respectively. In these studies, the authors described a wide range of biosensors, and different samples used for their validation. In particular, the authors of included studies defined their sensors as: *E.coli* whole-cell biosensors (63, 64), optical biosensors (61, 62, 66), surface plasmon resonance sensor (51), generic dipstick-based sensor (59), electrochemical biosensors (47, 49, 50, 55, 68, 70), biosensor based on aptamers (56, 57, 67), amperometric immunosensor (60), bioluminescent assays (52–54), colorimetric paper-based biosensor (65), biosensor based on proteins of prokaryotic origin (58), and highly sensitive square wave voltammetry (69). With respect to samples used for the development and/or validation of these biosensors, 5 studies regarded the development and application of biosensors in clinical (e.g., human blood and serum and urine samples) or bacterial samples (47, 50, 54, 60, 64), while 18 in environmental samples (e.g., milk and meat samples, animal feces and serum, and water samples) (48, 49, 51–53, 55–59, 61–63, 65–69).

**Table 3:** Summary of studies on biosensors for the detection of antibiotics in clinical or bacterial samples.

Authors and year of publications	Country	Type of sensor	Transduction systems	Sample	Target	Parameters
Rowe et al., 2010	USA	Electrochemical, ribonucleic acid aptamer-based biosensor	Electrochemical	Blood serum samples	To measure the concentrations of aminoglycoside antibiotics in human serum	Aminoglycoside concentrations range = 4–10 μg/ mL
Song et al., 2013	New Zealand	Genetically modified *E. coli* biosensor	Electrochemical	*E. coli* culture	To detect tetracycline using the bacterial respiratory gene, *nuoA*, as a reporter gene	LoD = 0.0028 μg ml^−1^
Tsang et al., 2014	China	Biosensor based on a fluorescein-labelled class C β-lactamase mutant	Optical	Bacterial culture	To develop a biosensor for the detection of cephalosporins and class C β-lactamase inhibitors	NA
Kling et al., 2016	Germany	Electrochemical microfluidic platform	Electrochemical	Plasma samples	To develop a platform useful for the surveillance and monitoring of antibiotics (tetracycline and streptogramin)	LoD = 6.33 and 9.22 ng mL^−1^ for tetracycline and pristinamycin, respectively
Yadav et al., 2020	India	Label-free amperometric biosensor	Electrochemical	Urine samples	To design a biosensor for detection of norfloxacin (NF) in human urine samples	LoD = 3.87 pM

**Table 4:** Summary of studies on biosensors for the detection of antibiotics in environmental samples.

Authors and year of publications	Country	Type of sensor	Transduction systems	Sample	Target	Parameters
Hansen et al., 2002	Denmark	*E. coli* whole-cell biosensor	Optical	Pig feces	To detect the concentration of chlortetracycline (CTC) and their correlation with tetracycline resistant bacteria	LoD = 0.03 mg/kg CTC
Weber et al., 2004	Switzerland	Biosensor based on proteins of prokaryotic origin	Optical	Milk and cow serum samples	To engineer prokaryotic antibiotic response regulators into a molecular biosensor configuration able to detect tetracycline, streptogramin, and macrolide antibiotics	LoD = 0.1–7.2 ng/mL, 2.7–70 ng/mL, and 1.7–5,000 ng/mL for tetracycline, streptogramin, and macrolide antibiotics, respectively
Caldow et al., 2005	UK	Optical SPR-based biosensor	Optical	Honey samples	To develop and validate an optical SPR-based biosensor for the detection of tylosin residues in honey	LoD = 0.5 μg kg^−1^
Link et al., 2006	Switzerland	Generic dipstick-based sensor	Optical	Serum, meat, and milk samples	To design an easy-to-handle dipstick-based assay for detection of antibiotic levels in serum, meat, and milk	LoDs = up to 40-fold below licensed threshold values in serum, meat, and milk
Lai et al., 2021	Australia	Biochemiresistor	Electrochemical	Milk samples	To develop a new type of biosensor for the detection of enrofloxacin	LoD = 2.8 pM
Cheng et al., 2014	China	Bioluminescent-bacteria-based assay	Optical	Milk samples; fish muscle; muscles, livers, and kidneys of cattle, chickens, and pig	To construct the *E. coli* pK12 harboring plasmid pRecAlux3 and develop a bioluminescent-bacteria-based assay for the detection of FQNs in animal-derived foods	LoD = 12.5–100 μg kg^−1^
Wang et al., 2016	China	Electrochemical aptasensor	Electrochemical	Milk samples	To design an electrochemical aptasensor for the detection of antibiotic residues based on target-induced and T7 exonuclease-assisted dual recycling signal amplification strategy	LoD = 4.0pM for ampicillin
Duyen et al., 2016	Japan	Colorimetric paper-based biosensor	Optical	Water samples	To develop a biosensor for the detection of antibiotics inhibiting bacterial protein synthesis, including aminoglycosides, tetracycline, chloramphenicol, and macrolides	LoDs = 0.5, 2.1, 0.8, and 6.1 μg/mL for paromomycin, tetracycline, chloramphenicol, and erythromycin, respectively
Kao et al., 2017	Taiwan	Live bacterial sensor strains integrated into a CCD-based lens-free optical analyzer (LumiSense)	Optical	Milk, egg white, and chicken essence, egg yolk samples	To detect antibiotic residues in food samples based on luminescence induction	LoDs = 8 ng/mL for milk, egg white, and chicken essence, and 64 ng/mL for egg yolk
Altintas et al., 2018	Germany	Nano MIP-SPR sensor	Optical	Milk samples	To extend the applications of nanoMIPs in food samples analysis to determine the presence of glycopeptide antibiotics in milk	LoDs = 4.1 ng mL^−1^ and 17.7 ng mL^−1^ using direct and competitive assays, respectively
Li et al., 2019	USA	Electrochemical biosensor based on hybrid nanowire/nanoparticle array	Electrochemical	Meat samples	To develop a biosensor for the simultaneously detection of penicillin and tetracycline and to validate it with real sample tests	LoDs = 10.5 μM for penicillin and 15.2 μM for tetracycline
Stevenson et al., 2019	USA	Affinity-based electrochemical biosensor	Electrochemical	Meat samples	To develop a biosensor for the detection of ceftiofur residues in meat samples	LoD = 0.01 ng/mL
Mohammad-Razdari et al., 2019	Iran	Pencil graphite electrode (PGE) modified with reduced graphene oxide (RGO) and gold nanoparticles (GNPs) for ultrasensitive detection of Penicillin G (PEN)	Electrochemical	Milk samples	To determine PEN in spiked milk from cow, sheep, goat, and water buffalo	LoD = up to 0.8 fM
Yazgan Karacaglar et al., 2020	Germany	Green fluorescence protein (GFP)-based bioassay	Optical	Milk samples	To develop a novel whole-cell based bioassay to be used for detection of some antibiotics	LoDs = 3.33, 0.29, 28.00, 618.36, and 33.17 μg/L for ampicillin, benzylpenicillin, gentamicin, neomycin, and tetracycline, respectively
Chinnappan et al., 2019	Saudi Arabia	Highly sensitive square wave voltammetry (SWV)-based sensor	Electrochemical	Water samples	To develop a voltametric aptasensor for the detection of azlocillin antibiotic	LoD = 1.2 pg./mL
Nag et al., 2021	India	Optical enzymatic biosensor	Optical	Milk, meat, and water samples	To develop and test a biosensor for the detection of β-Lactam Antibiotics in Food and Environment	LoDs = 0.18 nM in milk, 9 nM in chicken, and 0.18 nM in water
Liu et al., 2020	China	Aptamer modified SnO2/Bi2S3-based photoelectrochemical (PEC) sensor	Electrochemical	Milk samples	To develop a sensor for the detection of tobramycin (TOB) in milk	LoD = 4.28 nmol/L
Du et al., 2021	China	Lateral flow aptasensor	Optical	Water samples	To prepare DNA aptamer and develop lateral flow aptasensor combining recombinase polymerase amplification for the detection of erythromycin	LoD = 3.0 pM

#### 3.3.2. Biosensors in clinical or bacterial samples

In the context of the development of biosensors for detecting antibiotics in clinical samples, Kling and colleagues introduced a microfluidic platform capable of simultaneously conducting up to eight enzyme-linked assays with electrochemical readout. This advancement provides a versatile solution for efficient detection of multiple analytes in clinical settings. The authors devised microfluidic channel networks that encompassed separate immobilization sections, which could be individually activated for distinct assay procedures. The aim was the detection of two commonly employed antibiotic classes (i.e., tetracycline and streptogramin) in spiked human plasma, which was successfully done within a sample-to-result time of less than 15 min (50). Using urine samples, Yadav and colleagues described the development of an amperometric biosensor for the detection of norfloxacin. The biosensor was based on nanostructured yttrium oxide modified with chitosan. The electrode was modified using fluoroquinolones antibodies specifically targeting norfloxacin through covalent interaction. This modification led to a highly sensitive biosensor with a rapid response time of 10 min. The effectiveness of the biosensor was demonstrated by successfully recovering norfloxacin from spiked samples with a range of 90.5 to 101.1%. To validate the obtained results, the biosensor was compared with the enzyme-linked immunosorbent assay (ELISA), ensuring its accuracy and reliability. The findings were better – in terms of sensitivity and lower detection limit – than those obtained with existing ELISA, suggesting an interesting new way to develop sensitive and fast responsive biosensors and biochip devices (60).

In the field of medical analysis, RNA-aptamer-based biosensors have emerged as potential tools for point-of-care medical diagnostics. However, their susceptibility to nuclease degradation has posed a challenge. To address this issue and protect RNA-based biosensors, Rowe and colleagues have successfully developed an electrochemical sensor based on aptamers. This sensor specifically targets aminoglycosidic antibiotics in human serum, offering a robust and reliable platform for detection. The biosensor supported the rapid (<10 s) measurement of aminoglycoside concentrations over the therapeutically relevant range, with a complete process in less than 30 min. However, since it was quite vulnerable to degradation by nuclease, the authors reported that the ultrafiltration was necessary. Indeed, the ultrafiltration allowed the measurements in filtered serum calibrators, suggesting it as a possible general approach for the use of RNA probes (47).

With the goal of enhancing their previously developed biosensor, Tsang and colleagues focused on improving the stability and performance of the system for detecting cephalosporins and class C β-lactamase inhibitors. To address this, they introduced a Y150S mutation to suppress the hydrolytic activity of the fluorescein-labelled class C β-lactamase mutant (V211Cf), resulting in the construction of the biosensor Y150S/V211Cf. The findings demonstrated enhanced sustainability of the fluorescence signal and a rapid response specifically to cephalothin, a first-generation cephalosporin. As a result, this biosensor holds potential as a valuable tool for the rapid and specific detection of cephalosporins (54).

Additionally, Song and colleagues described a genetically modified *E. coli* biosensor to detect tetracycline using the bacterial respiratory gene, *nuo*A, as a reporter gene. The authors incubated the sensor cells with tetracycline at a relatively low temperature (15°C), aiming to reduce gene over-expression. Moreover, the authors used a low-copy number plasmid pBR322 to carry the trans-gene to reduce over-expression and to reduce background expression. As reported, the results showed an improvement in biosensor response (64).

#### 3.3.3. Biosensors in environmental samples

The misuse and overuse of antibiotics in non-medical settings, particularly in livestock farming to promote growth, has led to a rise in multidrug-resistant human pathogenic bacteria. Hansen and colleagues proposed an *E. coli* whole-cell biosensor for the detection of chlortetracycline in pig feces, and its correlation with tetracycline resistant bacteria. The tetracycline concentration was correlated with the appearance and maintenance of faecal coliform bacteria resistant to tetracycline. Notably, even as the concentration of tetracycline decreased, a high level of tetracycline resistance among the bacteria was observed and maintained (63).

As reported by Link and colleagues, European community legislators have restricted the veterinary use of antibiotics and banned them in stock breeding. To monitor antibiotic usage, the researchers developed a user-friendly dipstick-based assay capable of detecting antibiotic levels in serum, meat, and milk. This assay demonstrated detection limits that were up to 40 times lower than the officially licensed threshold values for various antibiotics, including tetracycline, streptogramins, and macrolides. The dipstick technology employed membrane strips coated with streptavidin and immobilized biotinylated operator DNA, which served as capture DNA to bind a bacterial biosensor containing a hexa-histidine (His6) tag. Upon exposure to specific samples, antibiotics triggered a dose-dependent release of the capture DNA, disrupting the interaction with the biosensor. The dipstick allowed for the determination of antibiotic concentration through visual readout. By dipping the strip into two different solutions, the conversion of a chromogenic substrate by a standard His6-targeted enzyme complex was initiated, leading to a colour change. The assay provided a rapid and sensitive detection method in a convenient format. Its application could be extended to monitor the unauthorized use of antibiotics, ensuring compliance with regulations and promoting responsible antibiotic usage (59).

Similarly, Weber and colleagues proposed a biosensor based on proteins of prokaryotic origin for the detection of tetracycline, streptogramin, and macrolide antibiotics in milk and cow serum samples up to 2 orders of magnitude below current European Commission threshold values. Aimed to optimize the biosensor for its use in a high-throughput-compatible ELISA-type format, the authors showed the conversion of the interaction between biosensor protein and DNA chemically linked to a solid surface into an immuno-based colorimetric readout correlating with specific antibiotics concentrations (58). Furthermore, Kao and colleagues observed values of ciprofloxacin below the minimal allowed values in milk, egg white, and chicken essence, and egg yolk. To achieve this goal, the authors proposed a method based on luminescence induction by live bacterial sensor strains integrated into a single windowless linear charge-coupled device (CCD)-based lens-free optical analyzer (LumiSense System). The biosensor featured an integrated system consisting of microfluidic sample chips, a bacterial incubation chip, and a linear CCD as the photodetection component. Luminescence emitted by the bacteria within the BacChip was captured by the CCD and recorded using PC-based software. The biosensor exhibited response times ranging from 20 to 80 min, and it only required basic processes such as mixing, dilution, and homogenization. This streamlined approach enabled efficient screening of food samples in a high-throughput manner (66).

Interestingly, Lai and colleagues introduced a biochemiresistor that utilized gold-coated magnetic nanoparticles, modified with antibodies, and magnetically assembled nanoparticle films over interdigitated electrodes, to detect enrofloxacin. The gold-coated magnetic nanoparticles were functionalized with anti-enrofloxacin IgM antibodies (Ab-Au@MNPs), which acted as biosensing particles. The surface-bound enrofloxacin molecules provided specific binding sites for the anti-enrofloxacin antibodies. When the biochemiresistor was immersed in a sample solution containing enrofloxacin, some of the anti-enrofloxacin antibodies dissociated from Ab-Au@MNPs and formed complexes with the enrofloxacin molecules in the solution. As a consequence, this brought the particles within the nanoparticle film into closer proximity, leading to a reduction in film resistance. Thus, greater amount of analyte, greater dissociation and lower resistance were contemporary observed. To validate the detection of enrofloxacin in real-life, the biochemiresistor sensor was tested with a complex matrix of milk, demonstrating a remarkable detection limit of 2.8 pM. These results validate the versatility of the biochemiresistor as a reliable tool that can be applied to various analytes simply by modifying the antibodies attached to the Au@MNPs. This flexibility and sensitivity make the biochemiresistor an attractive option for a wide range of applications (68). Considering that fluoroquinolones (FQNs) are extensively utilized as broad-spectrum antibacterial agents in animal husbandry and aquaculture, Cheng and colleagues developed a bioluminescent-bacteria-based assay specifically designed for detecting FQNs in animal-derived food products. This innovative approach aims to enhance the safety and quality control of such foods. Using the induction of the *recA*-promoter-fused luciferase reporter gene in *E. coli* pK12 harboring plasmid pRecAlux3, an SOS response by FQNs was evident. Particularly, the assay was able to recognize 11 FQNs – including the above-mentioned enrofloxacin – and to act on 11 tissues, including milk, fish muscle, and the muscles, livers, and kidneys of cattle, chickens, and pigs. The detection limits were between 12.5 and 100 μg kg^−1^, lower than the maximum residual limits. Indeed, the assay is considered as easy to perform, with a simple sample preparation method which only include the extraction, and accurate for the detection of FQNs in animal-derived foods (53).

To uncover antibiotic residues in food safety field, Wang and colleagues designed a homogeneous electrochemical aptasensor based on target-induced and T7 exonuclease-assisted dual recycling signal amplification strategy. The aptasensor was applied to assay ampicillin in milk, also by adding different amounts of ampicillin to demonstrate good anti-interference capability in complex matrix, and great potential in practical applications. In line, the detection limit obtained was down to 4.0 pM, superior to that of the reported literatures (55). In this context, other 4 studies detected antibiotic residues in milk, through the development of specific biosensors. Specifically, the research undertaken by Altintas and colleagues demonstrated the efficacy of a surface plasmon resonance sensor in detecting glycopeptide antibiotics in milk samples. The authors designed molecularly imprinted nanostructures with high affinity synthetic receptors, which were coupled with the proposed sensor, demonstrating an increased affinity between the receptors and the target molecule compared to natural receptors and other synthetic receptors. Indeed, the synthetic receptors coupled with the optical sensor can detect vancomycin in the range of 10–1,000 ng mL^−1^, with high sensitivity and selectivity (51). Within this context, Mohammad-Razdari and colleagues introduced an impedimetric aptasensor designed for detecting Penicillin G in cow, sheep, goat, and water buffalo milk, as well as water samples. The aptasensor employed a pencil graphite electrode that was modified with reduced graphene oxide and gold nanoparticles. By measuring the charge transfer resistance, the aptasensor achieved an impressive limit of detection as low as 0.8 fM. These results highlight the aptasensor as a highly sensitive, cost-effective instrument with great potential for early screening of food samples, specifically for the determination of Penicillin G (67). Similarly, Yazgan-Karacaglar conducted a study describing a novel bioassay that utilizes whole-cell organisms for detecting several antibiotics, including ampicillin, benzylpenicillin, gentamicin, neomycin, and tetracycline, in milk samples. In contrast to previous sensors, this bioassay employed *E. coli* cells expressing Green fluorescent protein (GFP) as the recognition agent. The inhibition of viable cells resulted in a decrease in fluorescence intensity, which served as a measure of inhibition rate. The limit of detection values for ampicillin, benzylpenicillin, gentamicin, neomycin, and tetracycline were found to be 3.33, 0.29, 28.00, 618.36, and 33.17 μg/L, respectively. These promising results support the adoption of this bioassay in the dairy industry and dairy husbandry sectors for the effective detection of antimicrobial contaminants (52).

Among aminoglycoside antibiotics, tobramycin extracted from the fermentation broth of *Streptomyces tenebrarius* has spectrum antibacterial activity and could be detected in milk. With this in mind, the study conducted by Liu and colleagues described a SnO2/Bi2S3-based photoelectrochemical aptasensor that, under optimal condition, reached a limit of detection of tobramycin in milk samples equal to 4.28 nmol/L. Indeed, the aptamer specifically captured tobramycin molecules, inducing a rise in electron transfer resistance and a subsequent decrease in photocurrent. Such high selectivity and sensitivity highlight the potential of this aptamer as a valuable on-field assay tool for food control (56).

Aside from milk, studies conducted by Li and Stevenson described the potential detection of antibiotics in meat samples. Specifically, Li and colleagues presented a novel electrochemical biosensor that utilized a hybrid nanowire/nanoparticle array integrated with biomolecular receptors for the simultaneous detection of penicillin and tetracycline. The electrode, consisting of an Au (L-cysteine) – Pt (penicillinase) nanowire array, exhibited excellent performance in terms of simultaneous detection and high sensitivity for both penicillin (41.2 μA μM^−1^ cm^−2^) and tetracycline (26.4 μA μM^−1^ cm^−2^). This innovative biosensor holds great potential for the rapid and accurate detection of these antibiotics in various applications. The real sample tests were conducted with chicken and beef extract, showing good recovery performance. Due to the unique structure of multisegmented and vertically aligned 3D nanowire array, the biosensor could represent an enhanced platform for simultaneous detection of various bioanalytes (48). In this context, Stevenson and colleagues proposed an affinity-based electrochemical biosensor for the detection of ceftiofur residues in meat samples. As reported, the biosensor used the electrochemical impedance spectroscopy to probe the binding between ceftiofur and sensor surface, which determined interfacial capacitive changes. The findings demonstrated the ability to detect ceftiofur within a short span of 15 min, even at low sample concentrations of 0.01 ng/mL in 1× phosphate-buffered saline. In addition, the biosensor exhibited a detection limit of 10 ng/mL in 220 mg ground turkey meat samples. These results highlight the biosensor’s effectiveness in accurately identifying ceftiofur in various sample types, making it a valuable tool for rapid and sensitive detection in food safety and veterinary applications (49).

In recent years, there has been an upsurge in the utilization of tylosin in apiculture, as bacterial brood illnesses have become resistant to oxytetracycline. The study conducted by Caldow and colleagues proposed the development and validation of an optical biosensor for the detection of tylosin residues in honey. As reported, the detection capability for tylosin was 2.5 μg kg^−1^, that was complementary to existing confirmatory methods. Due to fully automated system, the authors encouraged the use of this biosensor to extract and analyze up to 25 samples within a working day (62).

Using both food and water samples, Nag and colleagues described an optical enzymatic biosensor for the rapid and point-of-use detection of β-Lactam antibiotics such as penicillins and cephalosporins. In this study, the enzymatic hydrolysis of β-lactams led to changes in the polymeric structure of the nanofibers, resulting in an increase in evanescent wave absorbance. Notably, the biosensor demonstrated a minimal sample preparation requirement, only necessitating the removal of fat. To calibrate the sensors, ceftazidime was spiked into antibiotic-free milk, meat, and tap water samples. Intriguingly, the biosensor exhibited the ability to detect other β-lactam antibiotics, including ampicillin, amoxicillin, and cefotaxime. These findings highlight the versatility and potential of the biosensor for the detection of various β-lactam antibiotics in different sample matrices. The detection limits were 0.18 nM in milk, 9 nM in chicken and 0.18 nM in water. With a detection limit of 0.18 nM, the sensor could also detect β-lactam antibiotics in any wastewater sources. Similarly, the performance of the sensor was validated using a wastewater sample, and its efficacy was qualitatively analysed through high-resolution liquid chromatography coupled with mass spectroscopy. For these reasons, the biosensor could be useful for quality testing in food processing industries, but also for common effluent treatment plants to monitor the presence of β-lactam antibiotics in the influent and their removal in the treatment process (61).

Regarding various environmental matrices, three studies explored the potential application of biosensors for detecting antibiotics in water samples. In one study, Chinnipan et al. designed a highly selective and sensitive voltammetric aptasensor specifically for the detection of azlocillin, a broad-spectrum β-lactam antibiotic. This aptasensor demonstrated promising capabilities in accurately identifying and quantifying azlocillin in water samples, highlighting its potential for environmental monitoring and analysis. Using the systemic evolution of ligands by exponential enrichment method, the authors selected the DNA aptamers against azlocillin. Thus, the aptamer Az9 was used to construct the voltammetric aptasensor, which showed a limit of detection of 1.2 pg./mL for azlocillin. To achieve the quantification of the target antibiotic, a time span of 30 to 50 min was necessary. Notably, the sensor successfully confronted the task of directly detecting the target within intricate settings, including tap water and wastewater, achieving commendable recovery percentages (69).

Similarly, Du and colleagues proposed the preparation of DNA aptamers and the development of lateral flow aptasensor combining recombinase polymerase amplification for detection of erythromycin, which has polluted the aquatic environment for decades. The developed aptasensor achieved a remarkable detection limit of 3 pM for the target. Furthermore, when applied to real water samples, it effectively detected erythromycin at pM levels (57).

Moreover, Duyen and colleagues proposed a colorimetric paper-based biosensor for the detection of antibiotics inhibiting bacterial protein synthesis, such as aminoglycosides, tetracycline, chloramphenicol, and macrolides. By applying a water sample without antibiotics to the paper discs, the paper-based biosensor triggered a normal colour change from yellow to purple. This change was induced by b-galactosidase, which was synthesized on freeze-dried paper discs containing an *in vitro* transcription/translation system. However, in the presence of antibiotics, the colour change was hindered due to the inhibition of b-galactosidase synthesis. Notably, the paper discs exhibited the colour change within the temperature ranges of 15–37°C and pH ranges of 6–10. Furthermore, the paper-based biosensor demonstrated its capability to detect 0.5 mg/mL paromomycin in real environmental water samples using only the naked eye (65).

## 4. Discussion

Numerous strategies are in place to combat AMR. These encompass tracking antibiotic consumption, monitoring resistance trends among humans and animals, launching educational initiatives to encourage responsible antibiotic usage, and introducing antibiotic stewardship programs within healthcare systems. By enacting these holistic approaches, we can successfully confront the issues brought about by AMR and ensure the protection of public health (71, 72). Traditionally, the identification and measurement of bacteria have relied on labor-intensive procedures demanding specialized labs and costly equipment. Specimens like blood, saliva, urine, and food samples undergo assessment using different methods, including microscopic inspection, cell cultivation, biochemical and immunological assays, and genetic analysis. However, each technique has its limitations. While microscopy is rapid, its precision is questionable, as it necessitates bacterial growth on agar over several days and staining (73). Biochemical and immunological tests, like ELISA, have proven effective in pinpointing particular bacterial markers. Nonetheless, these tests often mandate skilled experts to carry out and accurately interpret the outcomes. The involvement of trained professionals is vital to assure the dependability and accurate analysis of these tests (74). Advanced genetic analysis methods such as PCR and Real-time PCR have facilitated bacterial strain identification. Nevertheless, both PCR and Real-time PCR are classified as expensive and intricate methodologies, relying on specialized equipment and the need for sample enrichment and purification before analysis (75). Another aspect to consider is the overuse of antibiotics in animals, where they are used both for therapeutic purposes and to enhance growth. This practice can lead to the presence of antibiotic residues in animal-derived food products, which poses a significant risk to human health. Addressing this concern necessitates vigilant monitoring of antibiotic residue levels in food products sourced from animals, including meat, poultry, dairy, eggs, and honey. Failure to do so could lead to a range of adverse outcomes, notably the potential emergence of AMR (76).

Within this intricate and multifaceted context, there has been a rising fascination with faster, economically efficient, and highly responsive techniques capable of detecting and characterizing bacteria, ARGs, and antibiotics in various clinical and environmental samples. This scoping review encompassed 48 studies detailing the development and validation of biosensors for identifying microorganisms (e.g., *S. aureus*, *M. tuberculosis*), ARGs (e.g., *mec*A in *S. aureus*, *rpo*B in *M. tuberculosis*), and antibiotics (e.g., β-lactams, quinolones, aminoglycosides, tetracyclines). The focus in microorganism and/or ARG detection primarily revolves around developing and validating biosensors for clinical and bacterial specimens. Conversely, the emphasis in antibiotic detection has primarily been on creating and validating biosensors for environmental samples. In addition to their target and sample types, the scoping review revealed diverse intrinsic characteristics among biosensors. Two primary classifications emerged: biosensors that necessitated sample processing (e.g., bacterial lysis) for detecting bacterial components like DNA and RNA, and processing-free biosensors enabling direct detection of whole bacteria without prior treatments (18). Categorization of biosensors can be extended based on the source of biorecognition probes, leading to two fundamental divisions: those arising from naturally existing elements (like enzymes and antibodies) and those deliberately conceived and constructed by humans (encompassing aptamers, molecularly imprinted polymers, and phage peptides). Enzymatic biosensors, where the enzyme either metabolizes the substrate or inhibits enzymatic reactions, and immunosensors utilizing antibody-coated sensors, have both showcased noteworthy specificity and cost-effectiveness. However, conventional biosensors have grappled with challenges related to storage, handling, and stability. On the contrary, engineered biosensors exhibit heightened affinity and specificity for bacteria, along with improved stability compared to components like enzymes or antibodies (77). Aptasensors, exemplifying this advancement, present distinct advantages over alternative affinity-based sensing techniques (78). Nonetheless, despite these strides, the field is still evolving, underscoring the necessity for further research and exploration.

Once the biosensing component identifies bacteria, the significance of transforming this interaction into a measurable signal through a suitable transduction system becomes evident. Our scoping review indicated that electrochemical (30, 36, 38–40, 43–50, 55, 56, 60, 64, 67–69), optical (22–24, 26–29, 31–35, 37, 42, 51–54, 57–59, 61–63, 65, 66), and mechanical (41) transducers were the most frequently utilized platforms for transduction in biosensing. Impedimetric and optical sensors were commonly adopted for detecting whole bacteria (73, 79), while the combination of diverse bioreceptors and transducers has led to an extensive spectrum of biosensors designed for the detection of complete bacteria (18). Mechanical biosensors usually operate by sensing alterations in the vibrational frequency of a piezoelectric crystal, enabling the identification of subtle mass changes, like the attachment of microorganisms onto a surface (80). Optical biosensors are widely utilized to perceive alterations in light characteristics generated by interactions between analytes and bioreceptors. They provide exceptionally precise and sensitive bacteria detection in a swift, real-time, and economical manner. Optical biosensors are classified into label-based approaches, including fluorescent techniques, and label-free methods. Particularly, plasmonic biosensors utilizing surface plasmon resonance (SPR) or surface-enhanced Raman spectroscopy (SERS) have been frequently employed for pathogen and antibiotic identification, as noted in our scoping review (35, 37, 51, 62). Nevertheless, electrochemical detection boasts numerous advantages over alternative analytical methods. It’s renowned for its cost-effectiveness, robustness, speed, and relative user-friendliness. Of particular note is its high selectivity in biological sensing, generating a signal based on electrochemical and physical alterations detected on a conductive polymer layer. Correspondingly, our comprehensive review encompassed a wide array of studies centered on developing electrochemical biosensors for identifying microorganisms, ARGs, and antibiotics (30, 36, 38–40, 43–50, 55, 56, 60, 64, 67–69). Electrochemical biosensors can be classified into three types according to the measured electrical parameter: (i) amperometric, (ii) potentiometric, and (iii) impedimetric. Among these, electrochemical impedance spectroscopy (EIS) stands out as the most frequently employed technique, falling under the impedimetric biosensor category. Diverse studies featured in the review (38, 39, 67) revealed that impedance alterations correlated with physicochemical changes contingent upon the interaction between the analyte and the bioreceptor. In this dynamic field, nanomaterials play a pivotal role in heightening sensitivity for target detection. Notably, materials like graphene-based substances, magnetic nanoparticles, and biocompatible metal/metal oxide nanoparticles (such as AuNPs) have found extensive utilization. Nanoparticles showcase exceptional optical and electrical properties, including heightened conductivity, photoelectrochemical activity, and an expansive surface-to-volume ratio. These attributes contribute to bolstered sensitivity and efficacy of biosensors in pinpointing specific targets. By crafting an optimal microenvironment, nanomaterials facilitate the retention of biologically active molecules and the generation of robust signal transduction, thereby magnifying the biosensor’s sensitivity (34, 35, 43, 44, 48, 67, 68). Recently, capacitive sensors, particularly interdigitated electrodes (IDEAs), have garnered considerable attention in the field. They emerge as a notably promising technology across various applications (40). In comparison to other impedance-based sensors, these transducers offer swift detection kinetics, a robust signal-to-noise ratio, rapid establishment of a steady-state response, budget-friendly costs, and straightforward miniaturization.

Several limitations should be considered when interpreting the outcomes of our scoping review. Our review possessed a wide-ranging scope, encompassing diverse research on biosensor advancement for identifying microorganisms, ARGs, and antibiotics across clinical, bacterial, and environmental samples. However, the possibility exists that pertinent studies indexed in alternative databases (e.g., Scopus or Google Scholar) might have been inadvertently omitted. Moreover, the timeframe covered by our scoping review could potentially have excluded recent articles, underscoring the ongoing evolution within the field. Consequently, forthcoming systematic reviews should contemplate the inclusion of more recent articles and a wider range of databases to attain a more comprehensive grasp of biosensors’ potential in the context of AMR. While numerous studies focused on biosensor development, the evaluation and validation of the described biosensors, especially through comparison with traditional molecular methods, were limited in number. Furthermore, there’s a call for additional research to ascertain whether the applicability of a biosensor in a specific matrix can be extended to other matrices of interest.

In conclusion, given the overarching differences in characteristics between different biosensors, it remains challenging to definitively pinpoint the superior or most poised biosensor for broad-scale implementation. Currently, research is exploring diverse paths to cater to varying research inquiries and demands. Moreover, the field continues to expand as new technologies are introduced, progressively enhancing precision, user-friendliness, and the potential for extensive adoption—elements that might have been lacking thus far. Nevertheless, certain overarching insights can be gleaned from these reflections. Biosensors have emerged as promising tools in the fight against AMR, ARGs, and antibiotic misuse. These devices offer a range of advantages, including their rapid detection capabilities, high sensitivity to trace amounts of substances, and specificity in targeting specific mechanisms, genes, or molecules associated with AMR. Biosensors enable real-time monitoring, simplifying on-site testing and reducing the need for extensive sample preparation. Their potential for miniaturization allows for portable devices, while some biosensors can even detect multiple targets simultaneously, increasing efficiency. By providing timely results, biosensors hold the potential to revolutionize the management of AMR, allowing for timely interventions and more effective patient care. However, the complexity of biosensor design and the challenges of validation against established methods are hurdles to overcome. Developing biosensors with high specificity and sensitivity often requires intricate engineering and rigorous optimization. Cross-reactivity risks must be addressed to ensure accurate detection, as biosensors might inadvertently respond to substances with similarities to the target. Additionally, the influence of sample matrix, such as environmental or clinical samples, can impact biosensor performance, demanding careful consideration during development and validation. While biosensors offer immense potential, there are practical considerations to bear in mind. Cost, for instance, can be a limiting factor, particularly for advanced technologies. Specialized expertise is needed for biosensor operation, interpretation, and maintenance. Regular calibration and maintenance are necessary to ensure accurate results over time, and ensuring user proficiency is crucial for consistent and reliable outcomes. Despite these challenges, biosensors hold immense promise in aiding the battle against AMR, offering a rapid, sensitive, and specific means of detection that could greatly impact public health strategies worldwide.

## Data availability statement

The original contributions presented in the study are included in the article/supplementary material, further inquiries can be directed to the corresponding author.

## Author contributions

RMSL, MB, AM, and AA: conceptualization. RMSL, MB, and AM: methodology. RMSL and AM: formal analysis and writing – original draft preparation. RMSL, MB, AM, MCLR, GF, and AA: investigation, and writing – review and editing. AA: supervision. All authors contributed to the article and approved the submitted version.

## Funding

This work has been partially funded by European Union (NextGeneration EU), through the MUR-PNRR project SAMOTHRACE (ECS00000022).

## Conflict of interest

The authors declare that the research was conducted in the absence of any commercial or financial relationships that could be construed as a potential conflict of interest.

## Publisher’s note

All claims expressed in this article are solely those of the authors and do not necessarily represent those of their affiliated organizations, or those of the publisher, the editors and the reviewers. Any product that may be evaluated in this article, or claim that may be made by its manufacturer, is not guaranteed or endorsed by the publisher.
